# The Avon Longitudinal Study of Parents and Children - A resource for COVID-19 research: Questionnaire data capture May-July 2020

**DOI:** 10.12688/wellcomeopenres.16225.2

**Published:** 2020-09-22

**Authors:** Kate Northstone, Daniel Smith, Claire Bowring, Nicholas Wells, Michael Crawford, Simon Haworth, Nicholas J. Timpson

**Affiliations:** 1 ALSPAC, Department of Population Health Sciences, Bristol Medical School, University of Bristol, Bristol, BS8 2BN, UK; 2 MRC Integrative Epidemiology Unit, Department of Population Health Sciences, Bristol Medical School, University of Bristol, Bristol, BS8 2BN, UK; 3 Bristol Dental School, University of Bristol, Bristol, BS1 2LY, UK

**Keywords:** ALSPAC, Children of the 90s, birth cohort study, COVID-19, coronavirus, online questionnaire, mental health

## Abstract

The Avon Longitudinal Study of Parents and Children (ALSPAC) is a prospective population-based cohort study which recruited pregnant women in 1990-1992 and has followed these women, their partners and their offspring ever since. The study reacted rapidly to the coronavirus disease 2019 (COVID-19) pandemic, deploying an online questionnaire early on during lockdown (from 9 ^th^ April to 15 ^th^ May). In late May 2020, a second questionnaire was developed asking about physical and mental health, lifestyle and behaviours, employment and finances.

The online questionnaire was deployed across the parent and offspring generations between the 26th May and 5 ^th^ July 2020. 6482 participants completed the questionnaire (2639 original mothers, 1039 original fathers/partners, 2711 offspring (mean age ~28 years) and 93 partners of offspring). 1039 new participants who did not respond to the first questionnaire deployed in April completed the second questionnaire.  A positive COVID-19 test was reported by 36 (0.6%) participants (12 G0 and 24 G1), 91 (1.4%; 35 G0 and 56 G1) reported that they had been told by a doctor they likely had COVID-19 and 838 (13%; 422 G0 and 416 G1) suspected that they have had COVID-19.

The observational data from both COVID questionnaires will be complemented with linkage to health records and results of biological testing as they become available. In combination, these data may help us identify true cases. Data has been released as an update to the original dataset released in May 2020. It comprises: 1) a standard dataset containing *all* participant responses to both questionnaires with key sociodemographic factors and 2) as a composite release coordinating data from the existing resource, thus enabling bespoke research across all areas supported by the study. This data note describes the second questionnaire and the data obtained from it.

## Introduction

Mitigation strategies against the coronavirus disease 2019 (COVID-19) pandemic in the UK escalated in March 2020, leading to a comprehensive set of restrictions on normal life (termed the ‘lockdown’) which took effect on March 23 ^rd^ 2020. Since then a gradual lifting of the restrictions has been introduced in England in particular. These include certain school years being allowed to return to school, groups of up to six people being allowed to meet outdoors (whilst adhering to social distancing), non-essential shops, pubs and restaurants re-opening and certain competitive sports returning behind closed doors ^1^. However, many normal activities remain restricted and the long-term impact of COVID-19 on employment, the economy and peoples’ livelihoods seems likely to be substantial. The COVID-19 pandemic and associated mitigation strategies will affect not just physical health in those who catch the virus, but also mental health, future employment, financial activity and personal relationships. It is therefore important for longitudinal population studies to continue to measure prospectively the impact of lockdown and its easing on their participants. Such studies are needed to understand the ongoing effects of mitigation strategies on health and well-being and identifying inequalities in response to the pandemic.

The Avon Longitudinal Study of Parents and Children (ALSPAC) is a unique three-generational study, comprising ‘G0’: the cohort of original pregnant women, the biological father and other carers/partners; ‘G1’: the cohort of index children; and ‘G2’: the cohort of offspring of the index children. The study has a wealth of biological, genetic and phenotypic data across these generations ^2– 5^. ALSPAC has been well placed to capture information across key parts of the population in light of the COVID-19 pandemic – in particular the contrast between those in higher risk (the G0 cohort; mean age ~59 years) and lower risk (the G1 cohort; mean age ~28 years) groups. We have been able to collect repeat data quickly using our existing infrastructure for online data collection.

The wider COVID-19 data collection in ALSPAC will include data from three main sources: self-reported data from questionnaires, data from clinical services based on linkage to medical and other records and information from biological samples. The data from these sources are intended to be complementary and help address different potential research questions around COVID-19.

This data note describes the data collected via our *second* online questionnaire between 26 ^th^ May and 5 ^th^ July 2020 and provides a summary of the participants who responded. The update to the original dataset obtained from our first online questionnaire ^6^ is described here, together with any variables that have been derived using both sets of questionnaire data. We also present a brief assessment of the factors associated with completing the first COVID questionnaire but not the second in this population.

## Methods

### Setting

ALSPAC is an intergenerational longitudinal cohort that recruited pregnant women residing in Avon, UK with expected dates of delivery 1 ^st^ April 1991 to 31 ^st^ December 1992 ^2, 3^. The initial cohort consisted of 14,541 pregnancies resulting in 14,062 live births and 13,988 children who were alive at 1 year of age. From the age of seven onwards, the initial sample was bolstered with eligible cases who had originally failed to join the study and there were subsequently 14,701 children alive at 1 year of age following this further recruitment ^4^. Please note, the study website contains details of all the data that is available through a fully searchable data dictionary and variable search tool.

In response to the COVID-19 it was necessary to develop a data collection strategy which was practical, would yield data quickly and could be updated and repeated if necessary. For these reasons, we chose to use an online only data collection approach for this, restricting our invites to those participants with a valid email address (and coordinated with a systematic communications/outreach campaign to obtain updated information from participants). The questionnaire was developed and deployed using REDCap (Research Electronic Data CAPture tools ^7^); a secure web application for building and managing online data collection exercises, hosted at the University of Bristol. The development of the first questionnaire is described elsewhere ^6^.

### Content design

Content for our second questionnaire was selected to address three needs:

1. The need to track changes in health and wellbeing over time using repeated measures. To address this, we repeated a panel of questions from our first questionnaire (e.g. mental health). By repeating questions, we are able to capture information about participants who did not complete the first COVID questionnaire.
2. The need to harmonize data collection with other cohorts to facilitate co-ordinated analyses. We addressed this by incorporating content from a coordinated and freely available COVID-19 questionnaire co-developed by ALSPAC. This questionnaire was developed in consultation with a network UK and international longitudinal population studies and partners through a process facilitated by Wellcome (see acknowledgements), which was still being developed at the time of the first COVID questionnaire.
3. The need to gather data to investigate specific hypothesis which could not be tested using the first questionnaire. These topics were suggested by our collaborators and included:
  • Food intake – Dr Laura Johnson, University of Bristol
  • Alcohol intake – Professor Matt Hickman, University of Bristol
  • Gambling – Professor Alan Emond, University of Bristol
  • Perception of risk – Professor Marcus Munafo, University of Bristol and Drs Alexandra Freeman and Sarah Dryhurst, University of Cambridge
  • Social contacts – Drs Amy Thomas and Ellen Brooks Pollock, University of Bristol

The questionnaire included 4 sections, and captured information on the following:

A. Seasonal symptoms
  • Symptoms of COVID-19 and negative control symptoms since mid-April 2020 (symptoms repeated from Q1)
  • Diagnosis with COVID-19 and resulting treatment
  • Whether participants were happy to be contacted about future research projects involving testing or taking biological samples

B. Behaviour as a result of COVID-19
  • Lifestyle changes since lockdown started
  • Food intake in the past month
  • Alcohol use (AUDIT-C; ^8^)
  • Gambling
  • Social contacts and methods of communication (repeated from Q1 with additional age groups)
  • Details of self-isolation (repeated from Q1)

C. Impact of the pandemic
  • Worries during the pandemic (repeated from Q1)
  • Depression assessed using the Short Moods and Feelings questionnaire (SMFQ; ^9^; repeated from Q1)
  • Anxiety assessed using the General Anxiety Disorder-7 questionnaire (GAD7; ^10^; repeated from Q1)
  • Well-being assessed using the Warwick-Edinburgh Mental Wellbeing Scales (WEMWBS; ^11^; repeated from Q1)
  • Perception of risk
  • Free text inviting participants to provide details of other ways they have been affected by the pandemic

D. About you and who you live with during the pandemic
  • Type and number of people participant lives with
  • Changes in living arrangements as a result of the pandemic
  • Postcode of current living location for geocoding purposes

E. Employment and finances during the pandemic
  • Employment prior to and during lockdown
  • Keyworker status and employment sector
  • Managing financially and claiming benefits
  • Food security

F. Children
  • Number of children and childcare support needs (G1 offspring with children enrolled in the ALSPAC-G2 study (children of the Children of the 90s ^4^) only)

We then provided a separate questionnaire for those G1 participants who had children enrolled in our G2 study. The contents and results of that additional questionnaire will be reported elsewhere.

The final questionnaire (REDCap PDF) used is available with the associated data dictionary (which includes frequencies of all variables that are available) and both are available as extended data ^12^.

### Invitation and reminder strategy

**Figure 1.**
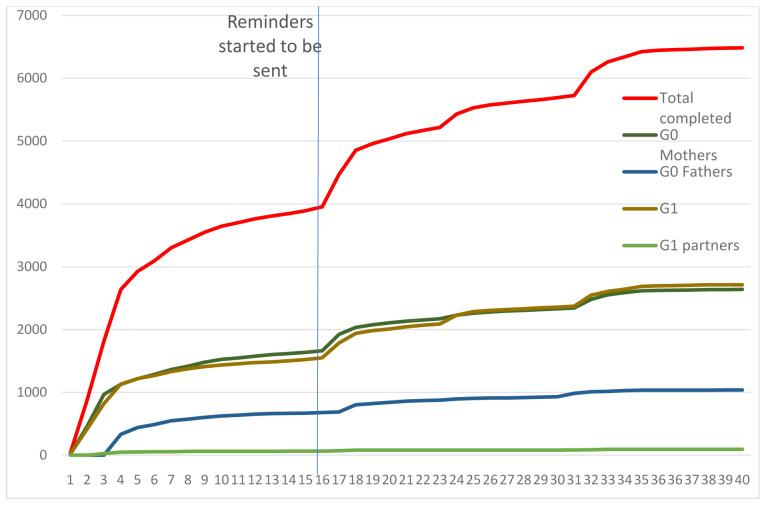
Completion rate by number of days second questionnaire was live.

Between the 26 ^th^ and 29 ^th^ May 2020, all participants (G0, G1 and G1 partners enrolled as part of G2 ^5^) for whom we had an active email address were sent an invitation to complete the questionnaire (n=12,560). Invites were tailored according to whether participants had completed the first questionnaire and according to gender, as we wanted to particularly encourage those who had not responded to the first questionnaire and males. On 10 ^th^, 19 ^th^ and 26 ^th^ June, a further 169, 119 and 50 additional invites were sent out respectively, as a result of outreach work undertaken by the ALSPAC team. Participants were not contacted if our administrative database record indicated that they were deceased, had withdrawn from the study, had declined further contact or had declined questionnaires. The questionnaire survey was live on the online platform for just over one month. On the 11 ^th^ and 12th June, any non-responders respectively were sent a reminder email to complete the questionnaire and a further text message was sent on 18 ^th^ June to those for whom we had a current mobile phone number. Finally, 1,972 dedicated reminders were sent on 26 ^th^ June to those participants who had previously completed our first COVID questionnaire but had not yet responded to the second. Compared to the first COVID questionnaire, we received a slower response to this questionnaire which required a different reminder process in order to increase participation (compare Figure 1 with the corresponding figure in our previous data note ^6^).

In addition, traditional (print, radio, tv) & social media (Facebook, Instagram and Twitter) were used to inform participants that the questionnaire was live, asking them to contact us if they had not received it and to encourage completion. These communication channels were also used to encourage re-engagement of friends and family back into the study. Unlike our standard questionnaires (usually completed annually) we did not provide any incentive for completion; however, we did offer a prize draw (three prizes of £100) for those who completed their questionnaire by 29 ^th^ June.

### Response rate

A total of 12,898 invitations were sent out and responses were received from 6482 participants (overall response rate of 50%) - see Figure 1.

As with our first COVID questionnaire, female participants were much more likely to respond than male participants. Table 1 summarises the response rate within each group organised by cohort structure. Response rate amongst G0s was similar for the second questionnaire compared to the first (57% vs 58%). A smaller proportion of G1 participants responded to the second compared to the first questionnaire (44% vs 51%). 1367 participants completed the first questionnaire but not the second and 1039 completed the second questionnaire but not the first. This means data is available from both questionnaires for 5443 participants in total.

**Table 1.** Number of participants who were eligible and who responded to the second COVID-19 questionnaire.

Cohort Group	Eligible ^1^	Responded to Q2 ^2^	Responded to Q1 and Q2
G0 Mothers	4646	2639 (57%)	2303 (50%)
G0 Fathers/partners	1821	1039 (57%)	871 (48%)
G1 Offspring daughters	3753	1914 (51%)	1575 (42%)
G1 Offspring sons	2395	797 (33%)	617 (26%)
G1 Offspring partners (female)	102	56 (55%)	49 (48%)
G1 Offspring partners (male)	181	37 (20%)	28 (16%)
**TOTAL**	**12898**	**6482 (50%)**	**5443 (42%)**

^1^valid email address, marked as contactable for questionnaires ^2^ Proportions of those invited (i.e. eligible)

### Key results

Characteristics of responders according to key variables that will be released with the complete dataset can be seen in Table 2. The population who responded were predominantly white (> 96%) and the majority had at least A-level qualifications (optional exams sat at the age of 18 years), with almost 80% of the G1 cohort in this category. Fathers were 3 years older on average than mothers (61.1 years vs 58.1 years) and G1 partners were two years older than G1 participants on average (29.8 years versus 27.8 years).

**Table 2.** Summary of key characteristics for those who responded; n (%) for categorical variables or mean (sd) for continuous variables.

	Mothers	Fathers/partners	Offspring	Offspring partners
Age (years)	58.1 (4.43)	61.1 (5.2)	27.8 (0.61)	29.8 (4.18)
Latest BMI ^1^	26.3 (5.07)	27.3 (3.97)	24.7 (5.24)	26.9 (4.99)
Latest Systolic BP ^1^	119.4 (14.1)	132.7 (13.36)	115.2 (10.92)	114.8 (12.53)
Latest Diastolic BP ^1^	70.5 (9.35)	77.2 (8.86)	66.8 (7.8)	65.7 (10.66)
Education level ^2^ ≥A level	1364 (54.1%)	693 (70.1%)	1662 (78.6%)	25 (59.5%)
Ethnicity ^3^ White	2477 (98.4%)	978 (98.9%)	2341 (96.6%)	Not available

^1^Data taken from the most recent clinic that individual attended where available ^2^Data taken from pregnancy questionnaires for G0 and from most recent questionnaire for G1 where available ^3^Data taken from pregnancy questionnaires for all

As with the first questionnaire, participants were asked whether they thought they have had COVID-19. Options were: ‘Yes, confirmed by a positive test’, ‘Yes, suspected by a doctor but not tested’, ‘Yes, my own suspicions’ or ‘No’. In the second questionnaire 36 (0.6%) respondents reported that they had tested positive to COVID-19, 91 (1.4%) reported that COVID-19 was suspected by a doctor but not tested and 838 (13%) believed they had COVID-19 due to their own suspicions. Table 3 summarises the responses to this question by cohort structure. Due to small numbers we have had to combine the first two categories together for the released dataset.

**Table 3.** Participant response to whether they have had COVID-19.

	G0 - parents	G1 – offspring (+partners)	Total
Yes, positive test	12 (0.3%)	24 (0.9%)	36 (0.6%)
Yes, doctor suspected, no test	35 (1%)	56 (2%)	91 (1.4%)
Yes, own suspicions	422 (11.5%)	416 (14.9%)	838 (13%)
No	3202 (87.2%)	2304 (82.3%)	5506 (85.1%)

Of the 5409 who completed this question in both questionnaires, with over 90% of people gave the same response in both questionnaires ( Table 4). There was a little more variability in the report of cases due to 'own suspicions', but this is not surprising given that since March 2020 there have been changes in the list of symptoms of COVID-19 in both official guidance in the UK and in media reports. Additionally, as the questionnaires were conducted approximately one month apart, it is possible that some participants became infected with COVID-19 after questionnaire 1 but before questionnaire 2; disagreement may not therefore necessarily reflect measurement error.

**Table 4.** Participant response to whether they have had COVID-19 in the first and second questionnaires (total n=5409).

Q2 response	Yes, positive test	Yes, doctor suspected, no test	Yes, own suspicions	No
Q1 response				
Yes, positive test	5	0	0	1
Yes, doctor suspected, no test	7	41	9	5
Yes, own suspicions	7	15	466	180
No	11	13	223	4426

As with the first questionnaire we applied the algorithm derived by Menni and colleagues ^13^ to predict ‘probable infection’ using data collected from an app-based symptom tracker ^14, 15^. This analysis was performed using Stata v15.0. This algorithm uses four symptoms: loss of smell and taste, severe or significant persistent cough, severe fatigue and skipped meals (coded as 1 if present and 0 otherwise), together with age and sex (1 male; 0 female). We had slight differences in wording and thus the algorithm (using the same weightings) applied was as follows:

-1.32 - (0.01 × age) + (0.44 × sex) + (1.75 × loss of loss of smell *or* taste)

+ (0.31 × *new* persistent cough) + (0.49 × severe fatigue)

+ (0.39 × decreased appetite). 

Predicted COVID-19 cases were obtained by applying an exp( *x*)/[1+(exp( *x*)] transformation and coding values >0.5 as cases. We applied this algorithm to the symptom data collected in questionnaire 2 which asked about symptoms experienced ‘since the middle of April (Easter Monday, 13 ^th^ April)’. Depending on when a participant responded this will have covered the last 6 to 10 weeks. In questionnaire 1 the proportion of predicted cases peaked in March with 4.19% of respondents predicted to be cases. All other months ranged between 2.10% - 2.38%. For this reporting period we predict that 3.1% of participants were possible cases (1.8% of female G0s, 3.2% of male G0s and 4.2% of G1 (3.8% of male G1 YPs and 4.3% of female G1 YPs). Our figures are still lower than the 5.36% of responders to the app ^13^ who were reported as likely being infected by the virus. 

Whilst we have presented these results as a mark of the type of analysis one can undertake using these data, we note that these predictions are subject to important assumptions, which we discussed in more detail in our previous data note ^6^. In summary: 1) the intercept term in the model, representing the *baseline risk of having COVID-19* is assumed to be the same in this population as in the Menni study population; 2) the slight difference in the wording of symptoms in our study are assumed to capture the same information as those in the Menni study and 3) the fixed effects in the model, representing the *association of these symptoms with COVID-19* is assumed to be the same here as in the Menni study population.

Of the 965 participants who reported definite or suspected COVID, only 8 stated that they had been admitted to hospital (2 female G0s, 2 male G0s and 4 G1s). However, it should be noted that four of these reported no overnight stay (2 G0s and 2 G1s). One participant reported admission to an intensive care unit and one other to a high dependency unit.

**Figure 2.**
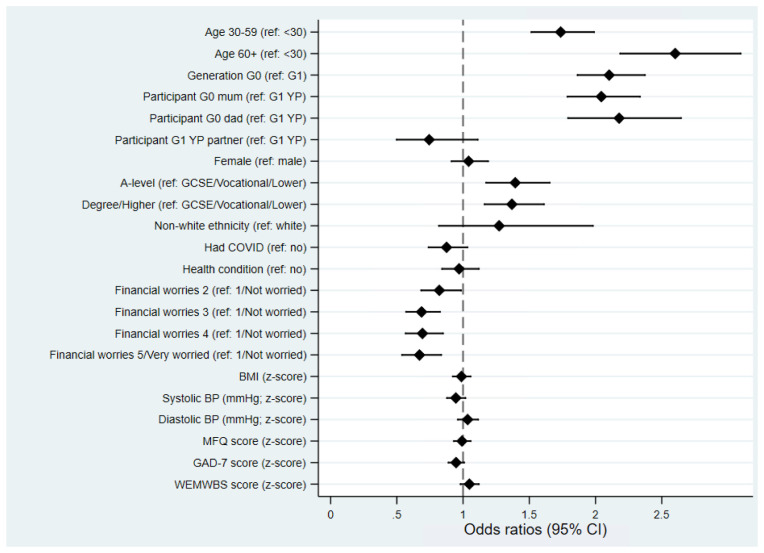
Forest plot describing the factors predicting completing the second COVID questionnaire, if the first was also completed. All results are odds ratios from logistic regression models with ‘completing questionnaire 2’ as the outcome. Other than ‘age’, ‘generation’ and ‘participant’ (which are univariable models), all models are adjusted for ‘generation’ (G0 vs G1). Results to the right of the dashed line indicate an increased odds of completing questionnaire 2 relative to the reference category, while results to the left indicate a decreased odds.

In order to assess potential reasons for non-completion of the second COVID questionnaire, which could bias comparisons between questionnaire waves, we explored whether any sociodemographic factors were associated with returning this questionnaire if the participant had returned the first COVID questionnaire ( Figure 2). Completing the second questionnaire was strongly associated with age/generation such that older/G0 participants were more likely to complete both questionnaires compared to younger/G1 participants. After adjusting for generation (G0 vs G1), response to the second COVID questionnaire was socially structured, such that those participants with higher education qualifications (a proxy for Socioeconomic position) were much more likely to complete the second questionnaire having completed the first. Greater financial worry at the time of the first COVID questionnaire was associated with an increased drop-out at the second time point. Finally, physical and mental health – including having COVID-19 – were not strongly associated with continued participation, although a weak effect of greater anxiety was associated with a decreased odds of continuing participation.

## Strengths and limitations of the data

This data collection has a number of strengths. Firstly, the timelines within which the collection occurred, allow comparisons between the stringent mitigation measures early on in the pandemic and later easing of the lockdown measures. Secondly, the availability of repeat data obtained over time throughout the pandemic along with pre-pandemic baseline measures allows assessment of longitudinal change in health or wellbeing. For example, we have already been able to demonstrate the short-term impact the pandemic has had on mental health ^16^. Thirdly, the alignment of measures with other UK studies provides potential for cross-cohort comparisons. This was achieved through the set of core questions developed by the Wellcome coordinated group ^17^ and has already facilitated a co-ordinated analysis of mental health measures in ALSPAC and Generation Scotland ^16^. We are planning further COVID-19 data collection towards the end of 2020 which will facilitate the examination of longer time impacts as these become clearer, particularly on the economy. Finally, we achieved an excellent response rate despite the lack of incentive and calling on our participants to take part in data collection for the second time in as many months. As we flagged for questionnaire 1, it should be noted that our online only strategy will likely have affected response rates from our G0 mothers who historically have tended to use paper questionnaires more than other sub-groups when completing questionnaires and for whom we are least likely to hold a current email address. However, the pandemic has led to a number of participants reaching out and getting in touch to provide these details, and indeed to re-engage with the study having dropped out previously. In addition, members of the study team have been contacting participants to ensure we have up to date email addresses, which goes partway to explaining the additional 1039 participants who completed this questionnaire but not the first.

A key limitation of this data collection is that in some cases, the data recorded is potentially identifiable. As with questionnaire 1 we have gone through each individual variable and made decisions as to whether we need to combine categories. This has only been carried out where we believe the data provides a high risk of potential disclosure (as detailed in the supplementary documentation file). Another limitation is that the response rate was non-random with regard to sex and socio-economic status; this could potentially introduce bias to analyses. Work is ongoing in the study to measure the potential selection bias that has been introduced in this and the first COVID-19 questionnaire and will be published in due course. We acknowledge there is a risk for people with severe COVID-19 to be under-represented in the study if they were too unwell to respond to questionnaires and we will be investigating this further using linkage to health records. Finally, we acknowledge that this observational dataset does not provide precise infection status. In particular, we note that: 1) asking individuals to report whether a doctor has suspected or whether they themselves suspected that they have been infected is insufficient to identify them as cases and 2) the use of the algorithm to predict cases is subject to a number of assumptions that we have discussed in the results section and elsewhere ^6^. The predicted case status also contains measurement error. We will address this by providing more accurate measures of COVID-19 status in the future using a combination of serological testing and data linkage.

In summary, data obtained in the second ALSPAC COVID questionnaire aimed to capture changes in many aspects of people’s lives as the pandemic persists and mitigation strategies continue to change. These data are available for researchers as described below.

## Consent

Completion of the questionnaire was optional and choosing to complete the questionnaire is considered informed consent for the questionnaire.

Ethical approval for the study was obtained from the ALSPAC Ethics and Law Committee and the Local Research Ethics Committees. Informed consent for the use of data collected via questionnaires and clinics was obtained from participants following the recommendations of the ALSPAC Ethics and Law Committee at the time. Study participants have the right to withdraw their consent for elements of the study or from the study entirely at any time. Full details of the ALSPAC consent procedures are available on the study website ^18^.

## Data availability

### Underlying data

ALSPAC data access is through a system of managed open access. The steps below highlight how to apply for access to the data included in this data note and all other ALSPAC data:

1. Please read the ALSPAC access policy ^19^ which describes the process of accessing the data and samples in detail, and outlines the costs associated with doing so.

2. You may also find it useful to browse our fully searchable research proposals database ^20^, which lists all research projects that have been approved since April 2011.

3. Please submit your research proposal ^21^ for consideration by the ALSPAC Executive Committee. You will receive a response within 10 working days to advise you whether your proposal has been approved.

Please note that a standard COVID-19 dataset will be made available at no charge (see description below); however, costs for required paperwork and any bespoke datasets required additional variables will apply.

### Extended data

Open Science Framework: ALSPAC COVID-19 First and Second Questionnaire. https://doi.org/10.17605/OSF.IO/GU35Y ^12^

This project contains the following extended data

1. ALSPAC COVID Q2 FINAL.pdf (The final questionnaire REDCap PDF)
2. ALSPAC_COVID_varlist.pdf (List of variable names and labels)
3. ALSPAC COVID Q2 data dictionary.pdf (Associated data dictionary including frequencies of all variables that are available)

Data are available under the terms of the Creative Commons Attribution 4.0 International license (CC-BY 4.0).

COVID-19 Questionnaire 2 Data File

Data from the second ALSPAC COVID-19 questionnaire is available in two ways.

1. A freely available standard set of data containing *all* participants together with key sociodemographic variables (where available) is available on request (see data availability section). This dataset also includes data obtained from the first COVID questionnaire. Subject to the relevant paperwork being completed (costs may apply to cover administration) this dataset will be made freely available to any bona fide researcher requesting it. Variable names will follow the format *covid2_xxxx* where *xxxx* is a four-digit number. A full list of variables released is available here: https://doi.org/10.17605/OSF.IO/GU35Y ^12^. Frequencies of variable and details of any coding/editing decisions and derived variables are also available in the data dictionary: https://doi.org/10.17605/OSF.IO/GU35Y ^12^
2. Formal release files have been created for G0 mothers, G0 fathers and G1 participants in the usual way and now form part of the ALSPAC resource (due to the small number of G1 partners contributing we will not be formally releasing this data, however, it may be available on request for specific G2 projects). These datasets (or sections therein) can be requested in the usual way. Variable names will replicate those in 1) above but as each variable in ALSPAC is uniquely defined we have added markers to denote the source of the variable. For example, in dataset 2, the age of the participant at completion (in years) is denoted by *covid2_9650*. In the mother’s dataset this will be denoted by *covid2m_9650*, for fathers/partner this will be *covid2p_9650* and for the G1 generation it will be *covid2yp_9650*. Frequencies for all variables for each participant group are available in the data dictionary in the usual way ^22^.

Text data and other potentially disclosive information will not be released until they have been coded appropriately. Table 5 describes the data that is withheld at the time of first release. Data will be incorporated back into both file sets as they become available.

**Table 5.** Data from questions that will not be released until coded.

Question number	Question text
**Section A**	
3	Date first told had COVID-19
5	Date first admitted to hospital for treatment of COVID-19
7	Date discharged from hospital for treatment of COVID-19
**Section B**	
4	Other form of gambling
7	Date started self-isolating Other reason for self-isolating
**Section C**	
1	What other reason causing worry
6	Is there anything else you would like to tell us about how the pandemic has affected you?
**Section D**	
2	Other change in living arrangements
3	Postcode (this is being geocoded and will otherwise only be available following our split stage protocol)
**Section E**	
1	Other employment situation
3	Other keyworker sector
4	Other keyworker sector person participant lives with is in
11	Other help given to someone
12	Other help received from someone
