# Supplementary material for: The Avon Longitudinal Study of Parents and Children - A resource for COVID-19 research: Questionnaire data capture May-July 2020
Source: Wellcome Open Res. 2020 Sep 22;5:210. Originally published 2020 Sep 9. [Version 2] doi: 10.12688/wellcomeopenres.16225.2 (PMC7512032; doi:10.12688/wellcomeopenres.16225.2)
Supplement: Peer review history

# Peer review: Reviewer response for version 2

**DOI:** 10.21956/wellcomeopenres.17942.r40554

**Published:** 2020-09-23

**Authors:** Denny Meyer

**Affiliations:** 1 Centre for Mental Health, Faculty of Health, Arts and Design, Swinburne University of Technology, Melbourne, Vic, Australia

Are sufficient details of methods and materials provided to allow replication by others?

Yes

Is the rationale for creating the dataset(s) clearly described?

Yes

Are the datasets clearly presented in a useable and accessible format?

Yes

Are the protocols appropriate and is the work technically sound?

Yes

Reviewer Expertise:

mental health during COVID-19 statistical analysis

I confirm that I have read this submission and believe that I have an appropriate level of expertise to confirm that it is of an acceptable scientific standard.

# Peer review: Reviewer response for version 2

**DOI:** 10.21956/wellcomeopenres.17942.r40555

**Published:** 2020-09-23

**Authors:** Crispim Cerutti Junior

**Affiliations:** 1 Departamento de Medicina Social, Programa de Pós-Graduação em Doenças Infecciosas, Universidade Federal do Espírito Santo (Ufes), Vitória, Brazil

The paper by Northstone *et al.* presenting the results of the application of questionnaires in two moments during the follow-up of the Avon Longitudinal Study of Parents and Children (ALSPAC) has received my previous review, which resulted in some criticism and suggestions. The authors incorporated most of my propositions and generated a more descriptive version of their manuscript. However, they opted to keep their original keywords, and did not extend their analysis to other aspects that I highlighted, as lifestyle, employment, and finances. I suggest to them also to avoid including brackets inside brackets, and initiating phrases by numbers.

Are sufficient details of methods and materials provided to allow replication by others?

Yes

Is the rationale for creating the dataset(s) clearly described?

Yes

Are the datasets clearly presented in a useable and accessible format?

Yes

Are the protocols appropriate and is the work technically sound?

Yes

Reviewer Expertise:

Infectious diseases and epidemiology.

I confirm that I have read this submission and believe that I have an appropriate level of expertise to confirm that it is of an acceptable scientific standard.

# Peer review: Reviewer response for version 1

**DOI:** 10.21956/wellcomeopenres.17821.r40376

**Published:** 2020-09-14

**Authors:** Crispim Cerutti Junior

**Affiliations:** 1 Departamento de Medicina Social, Programa de Pós-Graduação em Doenças Infecciosas, Universidade Federal do Espírito Santo (Ufes), Vitória, Brazil

The paper by Northstone *et al*. presents the results of the application of questionnaires in two moments during the follow-up of the Avon Longitudinal Study of Parents and Children (ALSPAC). The purpose was to verify the status of the mental health, lifestyle, employment and finances during the COVID-19 pandemic. However, the results are focused mainly on the estimation of a COVID-19 episode and the rate of questionnaires response. Despite the cohort itself representing a significant contribution to the study of the health status of the population, the approach reported in this paper has several limitations. At first, the response rate was low (50%), raising the possibility of a selection bias. Second, the criteria to establish the presence of the infection were mostly imprecise. Clinical suspicion or self-assessment is not enough evidence of the disease process. In third place, the authors gathered data about a positive test and a clinical suspicion, which have a substantial difference in their strength for evidence. The fourth limitation was the decision to apply the kappa index to verify the agreement in the information about the disease between the first and the second questionnaire. It is inadequate, as it would be expected the occurrence of the infection precisely in the interval between the two data collections. In the fifth place, another imprecise information rests on the estimation of the diseases based on an uncertain algorithm taking specific symptoms into account. I suggest to restrict the approach to the disease itself to a simple description of the answers, concentrating the analysis on the other issues intended to consist objectives of the study, i.e., mental health, lifestyle, employment and finances.

 Minor issue:

 Keywords: the authors should be compliant with MeSH. The following keywords are not in MeSh: ALSPAC, children of the '90s, birth cohort study, and online questionnaire.

Are sufficient details of methods and materials provided to allow replication by others?

Yes

Is the rationale for creating the dataset(s) clearly described?

Yes

Are the datasets clearly presented in a useable and accessible format?

Yes

Are the protocols appropriate and is the work technically sound?

Yes

Reviewer Expertise:

Infectious diseases and epidemiology.

I confirm that I have read this submission and believe that I have an appropriate level of expertise to confirm that it is of an acceptable scientific standard, however I have significant reservations, as outlined above.

# Response

**Published:** 2020-09-15

**Authors:** Kate Northstone

**Affiliations:** University of Bristol, UK

Thank you for the constructive comments on our data note. As a result, we have improved the clarity of our message; namely, that our data is observational and we can not claim ‘case’ status as a result. You noted five limitations and one minor issue in your review and we provide our response as follows:

1. The response rate was low (50%), raising the possibility of a selection bias.

We completely agree with this comment and work is ongoing in this area measuring the bias which is to be published separately. We have made more of this in the discussion adding the following: 

*“* *Work is ongoing in the study to measure the potential selection bias that has been introduced in this and the first COVID-19 questionnaire and will be published in due course.* *”*

 2. The criteria to establish the presence of the infection were mostly imprecise. Clinical suspicion or self-assessment is not enough evidence of the disease process.

Yes, we agree. The data note is providing a description of the responses we have received and as already noted we will be combining this data with linked health records and test results and to serology testing results in order to better establish ‘cases’. We have emphasised this self-reported data as a limitation in the discussion – please see our response to point 5 where we provide our overall discussion changes.

3. Third place, the authors gathered data about a positive test and a clinical suspicion, which have a substantial difference in their strength for evidence.

This is a fair point and as above we have enhanced the discussion in line with our response to point 2. We also make clear in the abstract that this data is observational and that we will use the questionnaire data in combination with linkage and biological testing to attempt to identify cases.

4. The decision to apply the kappa index to verify the agreement in the information about the disease between the first and the second questionnaire. It is inadequate, as it would be expected the occurrence of the infection precisely in the interval between the two data collections.

We have removed the kappa statistic. The aim of that analysis to determine crossover between the two points to check that if an individual reported infection in questionnaire 1 they would also report it in questionnaire 2. We already acknowledge that infection may have occurred in the interim but concede that kappa is not the best measure, as agreement between the time points is a little deceiving.

5. Another imprecise information rests on the estimation of the diseases based on an uncertain algorithm taking specific symptoms into account. I suggest to restrict the approach to the disease itself to a simple description of the answers, concentrating the analysis on the other issues intended to consist objectives of the study, i.e., mental health, lifestyle, employment and finances.

We agree with the reviewer that the algorithm is imprecise and had acknowledged this in the results section and in our previous data note. We have further emphasised the limitations of this and the general observational nature of the data in the discussion as follows:

*“…* *that this observational dataset does not provide precise infection status. In particular, we note that: 1) asking individuals to report whether a doctor has suspected or whether they themselves suspected that they have been infected is insufficient to identify them as cases and 2) the use of the algorithm to predict cases is subject to a number of assumptions that we have discussed in the results section and elsewhere ^6^* *”*

Finally, we have removed reference to the algorithm derived cases from the abstract and focussed only on the self-reported data here.

**Minor issue:** Keywords: the authors should be compliant with MeSH. The following keywords are not in MeSh: ALSPAC, children of the '90s, birth cohort study, and online questionnaire.

It is study policy to include ‘ALSPAC’ as a keyword and we have therefore kept this. We are happy to remove the other keywords as suggested and can replace with “Questionnaires”,

# Peer review: Reviewer response for version 1

**DOI:** 10.21956/wellcomeopenres.17821.r40375

**Published:** 2020-09-10

**Authors:** Denny Meyer

**Affiliations:** 1 Centre for Mental Health, Faculty of Health, Arts and Design, Swinburne University of Technology, Melbourne, Vic, Australia

Using a large sample of particpants from the Avon Longitudinal Study of Parents and Children this study reports the results of two questionnaires checking on COVID-19 symptoms and diagnoses. Interesting aspects of the study are a test of agreement for participants who completed both questionnaires and a comprehensive analysis of the factors associated with a lack of response for the second questionnaire. The algorithm used to estimate COVID-19 prevalence based on symptoms was clearly reported and the associated assumptions were clearly stated. The data provided and the detailed reporting of the methods used will allow replication by others. The authors are congratulated on their excellent response rate as well as an excellent report of the study methods and results.

Are sufficient details of methods and materials provided to allow replication by others?

Yes

Is the rationale for creating the dataset(s) clearly described?

Yes

Are the datasets clearly presented in a useable and accessible format?

Yes

Are the protocols appropriate and is the work technically sound?

Yes

Reviewer Expertise:

mental health during COVID-19 statistical analysis

I confirm that I have read this submission and believe that I have an appropriate level of expertise to confirm that it is of an acceptable scientific standard.

# Response

**Published:** 2020-09-15

**Authors:** Kate Northstone

**Affiliations:** University of Bristol, UK

Thank you for your positive review of our data note and for doing it so quickly!
